# Optimization of Mold Heating Structure Parameters Based on Cavity Surface Temperature Uniformity and Thermal Response Rates

**DOI:** 10.3390/polym17020184

**Published:** 2025-01-14

**Authors:** Xiaolong Qi, Jiaxing Li, Yingjie Liang, Zhonggui Xu, Yingru Li, Zhiyin Xie

**Affiliations:** 1 School of Intelligent Science and Engineering, Hubei Minzu University, Enshi 445000, China; 202330316@hbmzu.edu.cn (X.Q.); 202330315@hbmzu.edu.cn (J.L.); 202430322@hbmzu.edu.cn (Y.L.); 2019011@hbmzu.edu.cn (Z.X.); 2 Key Laboratory of Green Manufacturing of Super-Light Elastomer Materials of State Ethnic Affairs Commission, Hubei Minzu University, Enshi 445000, China

**Keywords:** rapid heating cyclic molding technology, parameter optimization, temperature uniformity

## Abstract

Rapid heating cycle molding technology has recently emerged as a novel injection molding technique, with the uniformity of temperature distribution on the mold cavity surface being a critical factor influencing product quality. A numerical simulation method is employed to investigate the rapid heating process of molds and optimize heating power, with the positions of heating rods as variables. The temperature uniformity coefficient is an indicator used to assess the uniformity of temperature distribution within a system or process, while the thermal response rate plays a crucial role in evaluating the heating efficiency of a heating system. The thermal response rate of the cavity and the temperature uniformity coefficient are set as optimization objectives to define parameter ranges for orthogonal experiments. The findings indicate that the optimal range for the lateral distance *L*_1_ is 20–30 mm, for *L*_2_ it is 50–70 mm, and for the vertical distance (*h*) it is 3–8 mm. The response surface multiple regression equation derived from the orthogonal experiment data demonstrates a model prediction error rate of 1.8% and 2.4%. Additionally, by applying particle swarm optimization to the regression equation, the study identifies an optimal scheme that reduces system energy consumption by 12.5%, achieves a thermal response rate of 0.75 k/s, decreases the temperature uniformity coefficient by 44.6%, and lowers the temperature difference by 52.17%. This optimization ensures efficient heating of the mold cavity, reduces energy consumption, and enhances the uniformity of the surface temperature distribution, ultimately improving the surface quality of the products.

## 1. Introduction

Injection molding is one of the most widely used processing techniques in the plastics industry. However, the poor surface quality performance of traditional injection molded products limits the application of high-gloss surfaces [1]. Additionally, it can lead to a range of defects in the final molded parts, including weld lines, dents, warping, sink marks, roughness, low gloss, and low replication accuracy [2,3,4,5,6,7,8]. To address these issues and enhance the surface quality of injection molded products, Rapid Heat Cycle Molding (RHCM)—a new injection molding process based on rapid heating and cooling—is gradually emerging as a replacement for traditional injection molding techniques. RHCM technology effectively eliminates surface defects like flow marks and weld lines. The rapid heating and cooling of the mold cavity surface enhances part quality but also increases production efficiency [9]. In RHCM, the process of rapid heating and cooling of the mold is crucial to ensuring product quality. Recent research has explored optimized heating methods in RHCM technology. Electromagnetic induction heating, which converts electrical energy into thermal energy through induction coils, offers advantages such as uniform heating, easy control, and a high degree of process automation [10,11,12,13,14]. However, it also presents challenges, such as high energy consumption, slower heating rates, and difficulties in mold design [15]. CHANG et al. [16] investigated the performance of infrared rapid surface heating injection molding using different types of reflectors, though this approach is only suitable for small parts with micro features [17]. WANG et al. [18] developed a steam-assisted RHCM technique, where high-temperature steam and cooling water are alternated through specialized valves to rapidly heat and cool the mold; however, this method is limited to simple parts like light guide plates.

Alternative methods, such as gas heating [19,20] and laser heating, are also limited in their ability to heat complex molds, highlighting the need for practical and versatile heating solutions. Among these, electric heating rods have been successfully applied in engineering contexts [21], becoming a key method in RHCM technology. However, uneven temperature distribution can arise from variations in the placement and power of heating rods within the mold, leading to inconsistent surface quality and potentially resulting in high thermal stress and significant product deformation [22].

To address the issues caused by uneven temperature distribution on the cavity surface, researchers worldwide have extensively studied the factors influencing cavity temperature distribution. WANG et al. [23] proposed an innovative “bench” structure with an elevated coupler bolt design, which significantly reduces sink marks on the outer surface of parts during RHCM processing. WANG et al. [24] introduced baffles into the heating/cooling channels, improving both the efficiency and uniformity of mold heating and cooling.

While RHCM technology offers various heating methods, detailed studies on cavity surface temperature distribution and its influencing factors under rapid temperature gradient conditions remain scarce [25]. To produce high-gloss, weld-line-free plastic parts and enhance production efficiency, WANG et al. [26] proposed a novel RHCM mold structure with a floating cavity, aimed at improving heating/cooling efficiency and temperature uniformity. Li et al. [27] applied finite element analysis and a Pareto-based genetic algorithm to optimize the mold, achieving more uniform temperature distribution on the fixed mold insert cavity surface. WANG et al. [28] experimentally evaluated the heating efficiency, temperature uniformity, and structural strength of a floating cavity and optimized the cavity structure by integrating response surface models with a particle swarm optimization algorithm.

The exploration of rapid heating methods and optimization of heating parameters in RHCM can be traced back to 2006 [9], In recent years, this technology has made further progress based on various heating methods. Berlin [29] investigated a mold heating method using infrared radiators, which offers advantages in terms of both the achieved temperature and cost efficiency. Mrozek [30] studied and optimized the heating process by examining factors such as the configuration of the inductors and magnetic concentrators in the induction heating method, thereby improving heating efficiency. The In-GMTC method [31,32] is a gas-assisted temperature control heating technique that uses internal gas to regulate the thickness of mold inserts and the gas temperature at different levels, enabling rapid control of the mold surface temperature. This gas heating method is applicable to nearly all mold materials and has the added advantage of preventing mold overheating. Kitayama [33] developed a heater-assisted RHCM technology that successfully reduces weld lines and shortens cycle time. Lee et al. [34] proposed a multi-layered mold structure that uses a carbon nanotube (CNT) network film as the heat source for the RHCM process. This method eliminates the width and depth of weld seams in the molded product, effectively improving the gloss and surface quality of the product compared to traditional methods. The latest advancements in heating methods for RHCM technology have reached a relatively mature stage but still exhibit some engineering shortcomings. For instance, the induction heating method is only suitable for steel molds with high magnetic permeability and is prone to overheating. The heater-assisted RHCM method still faces issues such as low heating efficiency. Infrared radiation heating, gas-assisted heating, and CNT network film heating methods come with high costs for peripheral equipment. The electric heating rod mold cavity heating method is a commonly used heating technique in engineering, and has been applied in the injection molding industry for over a decade. While it is not as efficient as methods like infrared or induction heating in terms of rapid heating, it offers higher stability and reliability. Its mature process makes it more flexible and cost-effective, capable of being applied to the heating of most mold materials. By further optimizing and studying the heating process of this mature heating technology, it is possible to improve product heating quality and efficiency, while also providing insights for other heating methods in RHCM technology.

Despite the progress in optimizing rapid heating methods in RHCM technology, there is limited research focused on enhancing cavity temperature uniformity while maintaining the thermal response rate. Few studies systematically investigate the factors affecting mold temperature. This paper employs numerical simulation to analyze the thermal response rate and temperature distribution of the mold cavity. By examining the effects of mold parameters, and using orthogonal experiments and particle swarm optimization algorithms, we conduct multi-objective optimization of the thermal response rate and temperature uniformity of the cavity based on factors such as heater position parameters and heating power, with results analyzed accordingly.

## 2. Numerical Modeling of the Mold Heating Process

### 2.1. Mathematical Model

The transient heat transfer process in an electrically heated cavity plate involves heat generation through the resistance heating of electric heating rods. Heat is conducted through contact with the mold wall and subsequently transferred to the cavity surface, achieving rapid and stable heating. Thus, the mold heating process is a three-dimensional, unsteady heat conduction process with an internal heat source.

The heat conduction rate equation can be expressed as:

(1) $$q=-\lambda \frac{\partial T}{\partial n}$$

In this equation, q denotes the heat flux density in W/m^2^, λ represents the thermal conductivity in W/(m·°C), and $\frac{\partial T}{\partial n}$ indicates the temperature gradient in the n-direction, with the negative sign showing the direction.

By applying Fourier’s law of heat conduction, the principle of energy conservation, and the three-dimensional unsteady-state heat conduction partial differential equation in a Cartesian coordinate system, the heat conduction differential equation for the mold’s electric heating process can be derived as follows [35]:

(2) $$\frac{\partial T}{\partial t}=\frac{\lambda }{\rho c}\left(\frac{\partial^{2}T}{\partial x^{2}}+\frac{\partial^{2}T}{\partial y^{2}}+\frac{\partial^{2}T}{\partial z^{2}}\right)+\frac{\Phi }{\rho c}$$

In this context, T represents the temperature in Kelvin (K); t denotes time in seconds (s); λ is the thermal conductivity of the mold material in W/(m·K); ρ is the density of the mold material in kg/m^3^; and Φ represents the heat generated by the internal heat source per unit volume per unit time, measured in W/m^2^.

### 2.2. Geometric Modeling

**Figure 1:**
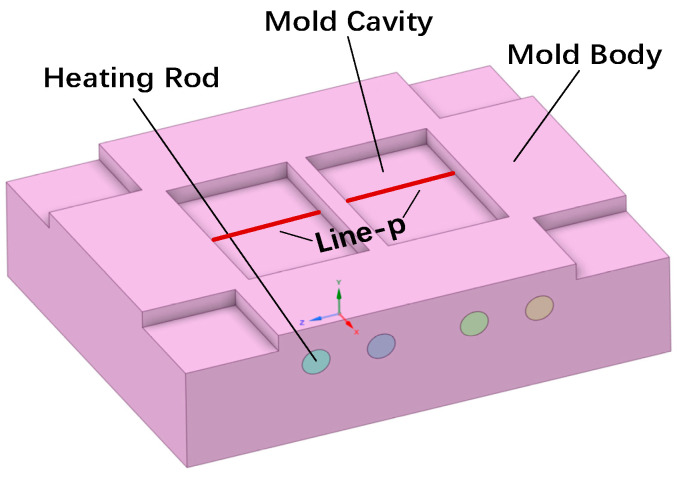
Model structure of the mold.

The model structure discussed in this paper, as shown in Figure 1, is the primary mold of an injection mold, consisting of the mold body, cavity, heating rods, and mold base. To achieve more uniform heating of the cavity surface in the RHCM process, the mold uses a symmetrical dual-module structure.

The mold dimensions are 260 mm in length, 208 mm in width, and 70 mm in thickness, with a cavity size of 96 mm × 65 mm × 10 mm. The mold is heated using cost-effective, high-efficiency electric heating rods [22]. To ensure rapid and uniform heating of the entire mold cavity, four heating rods with a diameter of 15 mm are used, spaced 53 mm apart and positioned 12.5 mm from the cavity surface. The power of each heating rod is 800 W. A characteristic line, Line-P, is placed at the center of the cavity surface for temperature measurements.

**Figure 2:**
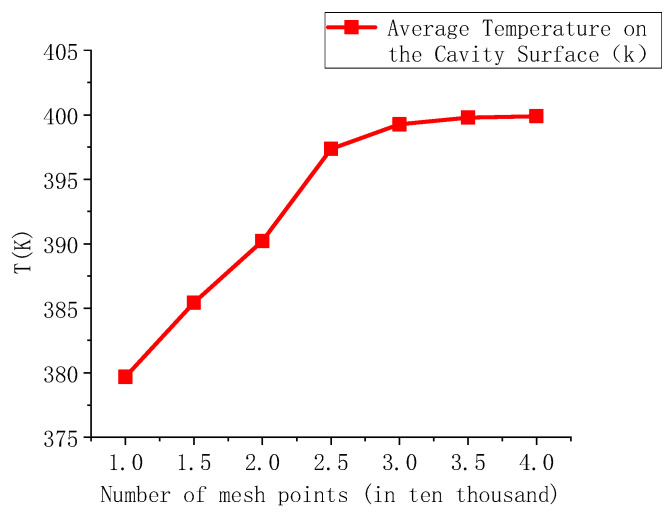
The relationship between the number of grids and the average temperature on the cavity surface.

Mesh generation is a critical step in simulation modeling. High-quality meshes, however, often come with increased computational costs. Therefore, finding an effective meshing strategy can minimize data exchange time while ensuring mesh quality, thus improving computational efficiency [36]. To obtain more accurate results, a hexahedral meshing method is used to discretize the model structure. The mesh is refined on the mold cavity surface and the heating rod region, with additional refinement applied to key areas. This refinement significantly enhances the accuracy of the data without excessively compromising computational efficiency. Based on these objectives, seven mesh scales were constructed, with the mesh count ranging from 10,000 to 40,000 elements. The impact of mesh resolution on the average temperature of the cavity surface was then assessed. The relationship between mesh count and average surface temperature is shown in Figure 2. When the mesh count exceeds 35,000, the temperature difference is only 0.1%, and the mesh quality remains satisfactory. Therefore, a mesh count of 35,000 was chosen for the simulation tests in this study.

### 2.3. Physical Properties and Boundary Conditions

The thermal operating parameters of the injection mold and electric heating materials are presented in Table 1. Given that the changes in operating parameters within the 300–500 K range are minimal, constant thermal parameters are used. C, the specific heat capacity, is measured in J/(kg·K).

Boundary Conditions: Based on actual operating conditions, the initial temperature of the mold is set to 303 K. The cavity surface, which is part of the injection area, is also set as an adiabatic surface. The lower surface of the mold body, in contact with the mold base, is set as an adiabatic surface. The four surfaces in contact with air are set as convective boundary conditions, and the formula for calculating the convective heat transfer coefficient is as follows [37]:

(3) $$\bar{h}=\frac{{\bar{Nu}}_{L}\cdot k}{L}$$

${N_{u}}_{L}$ is the Nusselt number, *K* is the thermal conductivity of the fluid, and *L* is the characteristic length. The Nusselt number calculation formula in the vertical direction is as follows:

(4) $${\bar{Nu}}_{L}={\left\{0.825+\frac{0.387Ra_{L}^{1/6}}{{\left[1+{\left(0.492/Pr\right)}^{9/16}\right]}^{8/27}}\right\}}^{2}$$

$Ra_{L}$ is the Rayleigh number. The Nusselt number calculation formula in the horizontal direction is as follows:

(5) $${\bar{Nu}}_{L}=0.54Ra_{L}^{1/4}$$

**Figure 3:**
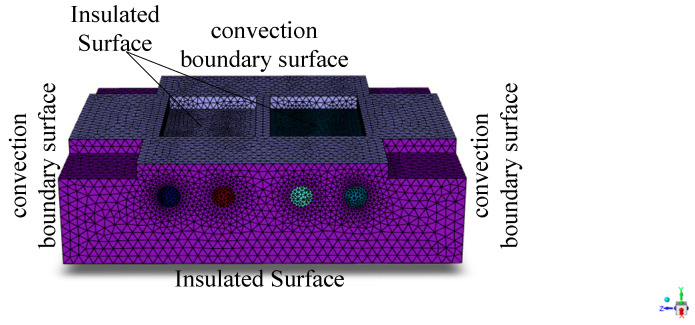
Schematic diagram of mold thermal boundary conditions.

The average convective heat transfer coefficient for the four surfaces in contact with air, obtained through calculation, is 20 W/m^2^K. The ambient temperature is 300 K, and the heating process of the heating rod is modeled as an internal heat source within the volume. Figure 3 shows a schematic diagram of the thermal boundary conditions of the mold.

## 3. Mold Cavity Temperature Distribution Analysis

### 3.1. Cavity Temperature Analysis

**Figure 4:**
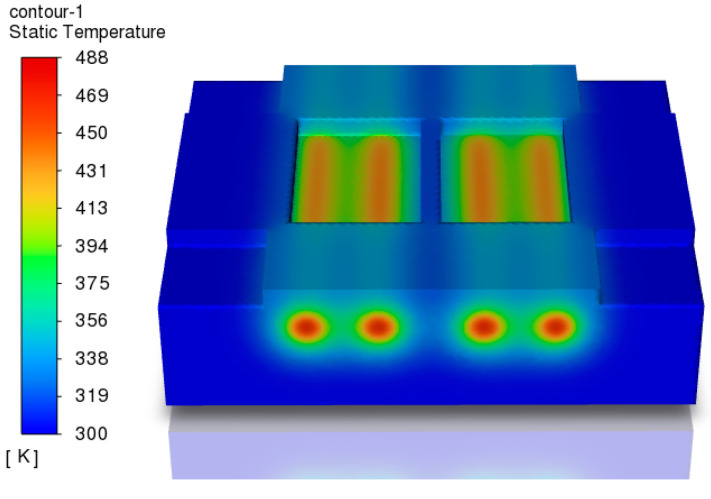
The temperature distribution map of the mold after heating.

In this study, ANSYS software 2022 R1 (ANSYS, Inc., Canonsburg, PA, USA) was employed to solve the energy equation for the transient heating process of injection mold cavities, The temperature distribution of the mold after heating and the temperature distribution on the cavity surface were calculated through simulation. The temperature distribution of the mold at the end of the heating process is shown in Figure 4. The cavity temperature rises significantly, exhibiting a high-temperature region along the direction of the heating rod and a clear temperature gradient distribution. The temperature in the high-temperature region begins to decrease as it moves away from the heating rods, reaching a steady state at both ends of the cavity.

**Figure 5:**
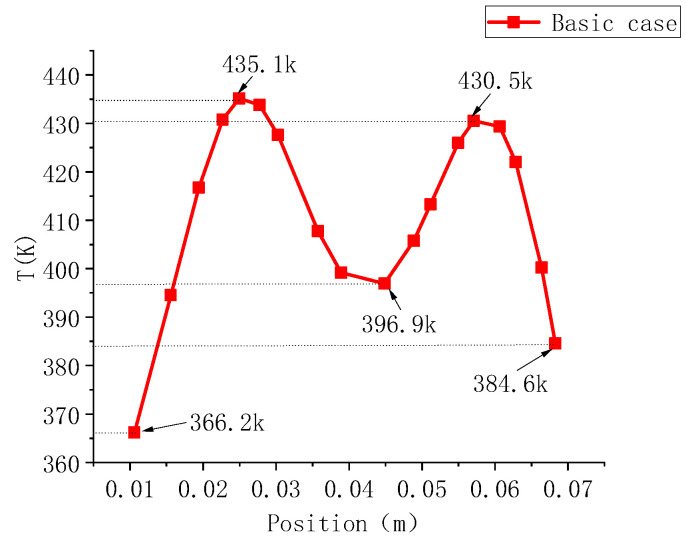
Temperature distribution map of the cavity surface after heating (Line-P).

Additionally, the cavity temperature distribution along Line-P is illustrated in Figure 5. The high-temperature phenomenon on the cavity surface is mainly concentrated near the heating rods, with peak temperatures of 435.1 K and 430.5 K. The temperature then rapidly decreases on both sides of the peaks, eventually dropping to a minimum of 396.9 K between the heating rods. At both ends of Line-P, the temperature decreases to 366.2 K and 384.6 K, respectively, resulting in a maximum temperature difference of 68.9 K, highlighting significant non-uniformity in the cavity surface temperature. Such uneven temperature distribution can lead to inconsistencies in the crystallinity of polymer materials and uneven cooling shrinkage, resulting in deformation of plastic products. Therefore, further analysis and optimization of factors affecting the cavity surface temperature distribution are crucial.

### 3.2. Experimental Validation

**Figure 6:**
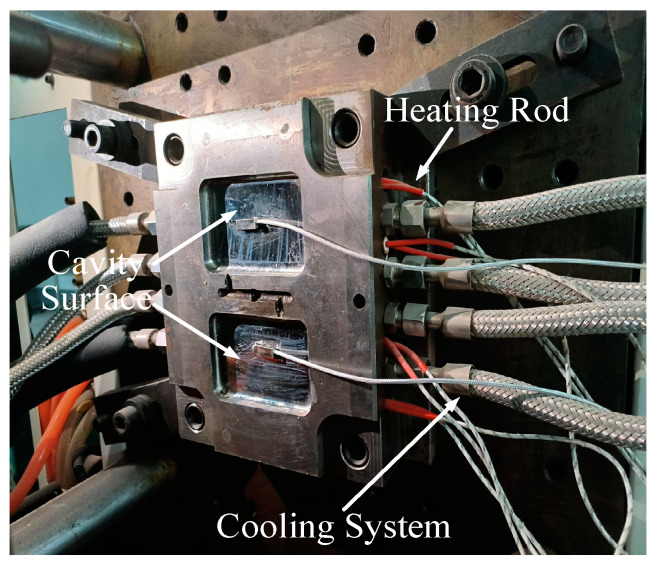
Experimental equipment.

**Figure 7:**
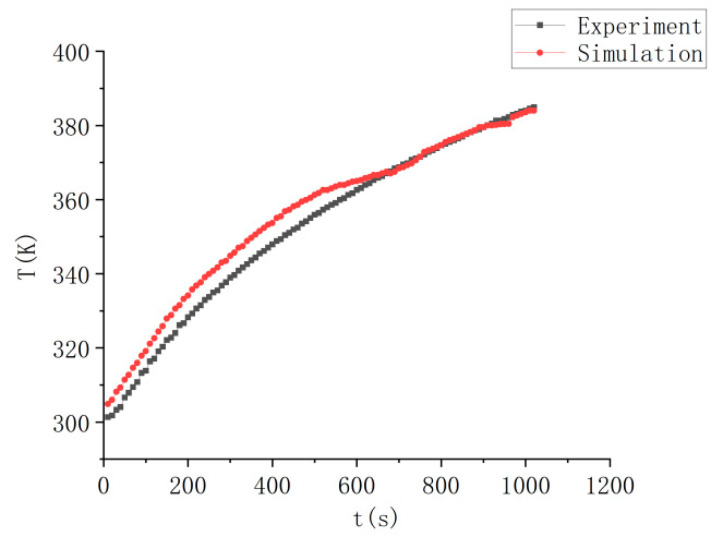
Experimental verification of data controllability.

This study conducted experiments using laboratory injection molding equipment and molds, maintaining parameters consistent with the simulation conditions. The experimental setup is shown in Figure 6. A comparative analysis of experimental and simulation data for the heating process revealed a close alignment in temperature distribution curves. The experimental verification of data controllability is shown in Figure 7. In the figure, the temperature rise trend of both the experimental and simulation data is consistent before 600 s, with the error likely due to the gap between the heating rods and the mold. The relative error is less than 8%. After 600 s, the numerical trends of the two datasets are in good agreement. This confirms the reliability of the numerical simulation, establishing a robust foundation for optimization across various parameter conditions in this study.

## 4. Design of Cavity Temperature Uniformity Optimization Scheme

### 4.1. Evaluation Criteria

Cavity temperature uniformity and the thermal response rate are key parameters in this study [17]. The temperature uniformity of the cavity surface during mold heating directly determines product quality. Uneven temperature distribution can result in excessive temperature differences on the surface or inside the product, leading to deformation, cracking, or other quality issues. The thermal response rate directly affects production efficiency, and optimizing this rate helps in designing more efficient heating systems. The temperature uniformity is evaluated using the uniformity coefficient, $U_{T}$:

(6) $$U_{T}=\sqrt{\frac{{\left(T_{1}-\bar{T}\right)}^{2}+{\left(T_{2}-\bar{T}\right)}^{2}+\ldots +{\left(T_{n}-\bar{T}\right)}^{2}}{n}}$$

where *n* is the number of test points, *T* represents the temperature at each test point (unit: K), and $\bar{T}$ is the average temperature of those test points (unit: K).

The evaluation criterion for thermal response rate $R_{heating}$ is:

(7) $$R_{heating}=\frac{T^{\prime }-T_{0}}{\Delta t}$$

where *T*_0_ is the initial average cavity temperature, *T*′ is the average cavity temperature at the end of heating, and Δt is the heating duration for the mold (in s).

In RHCM, heating the molding surface to 100 °C can completely prevent the formation of a frozen layer, contributing positively to reducing product defects [3] and enhancing part appearance [18]. Therefore, the target average cavity temperature after heating is set to 375 K, with an initial mold temperature of 300 K and a heating duration of *T* = 100 s. Consequently, the cavity thermal response rate should exceed 0.75 K/s. Additionally, a lower temperature uniformity coefficient indicates more uniform cavity surface temperatures, reflecting improved optimization results.

### 4.2. Optimization Parameter Range

**Figure 8:**
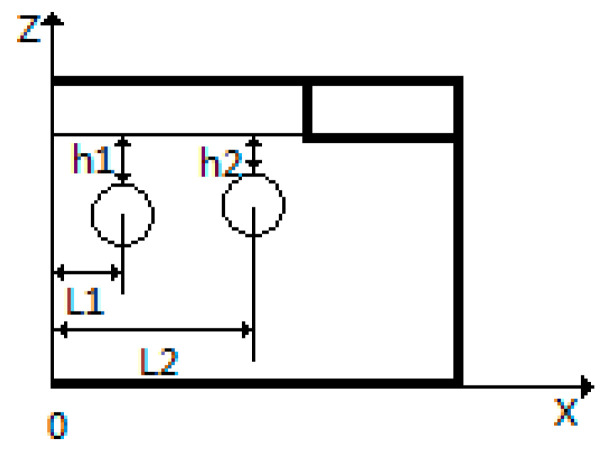
Schematic of mold heating rod installation dimensions (symmetrical structure).

Based on the cavity surface temperature distribution map in Figure 4 and the temperature distribution data in Figure 5, it can be concluded that the horizontal and vertical positions of the heating rods significantly affect the temperature distribution on the cavity surface. Therefore, the optimization parameters include the positions of the heating rods, *L*_1_, *L*_2_, *H*_1_, and *H*_2_, as illustrated in Figure 8. The power levels for the four heating rods, *q*_1_, *q*_2_, *q*_3_, and *q*_4_, can be adjusted within a range of 400 to 1200 W.

To minimize the temperature uniformity coefficient while ensuring the required cavity thermal response rate, a large-scale optimization process is necessary. To control the number of subsequent orthogonal experiment trials, the range of values for the optimization variables must be narrowed. The specific optimization plan is outlined in Table 2.

The numerical simulation analysis of the 12 schemes above shows the cavity thermal response rates for each scheme, as presented in Table 3. The horizontal distances of heating rods 2 and 3 have minimal impact on the cavity thermal response rate, while the horizontal distances of heating rods 1 and 5 have a more significant effect. The vertical distances of the heating rods have a notable influence on the cavity thermal response rate.

**Figure 9:**
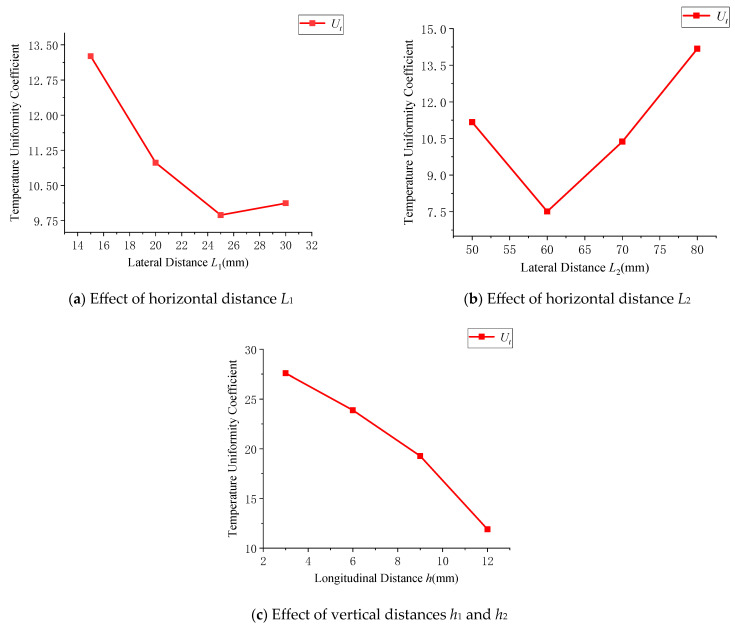
Impact of horizontal and vertical distances on the temperature uniformity coefficient.

The effect of changes in the horizontal and vertical distances of the heating rods on the cavity surface temperature uniformity coefficient is shown in Figure 9. For temperature uniformity, the optimal range for the horizontal distance *L*_1_ is between 20 mm and 30 mm, while for *L*_2_, the optimal range is between 50 mm and 70 mm. The vertical distance has a linear relationship with both temperature uniformity and thermal response rate. Considering only the effective value range under the minimum thermal response rate condition (0.75 K/s), the range for h is between 3 mm and 8 mm. Similarly, the heating rod power (*q*) is set within the range of 600 W to 900 W.

Within the operating range of the mold, the cavity surface temperature demonstrates a good thermal response rate and temperature uniformity, effectively enhancing the temperature distribution uniformity in the mold cavity. This establishes a range of operating parameters for the further design and optimization of target parameters in subsequent stages.

## 5. Cavity Temperature Uniformity Optimization Study

### 5.1. Target Optimization Process Design

**Figure 10:**
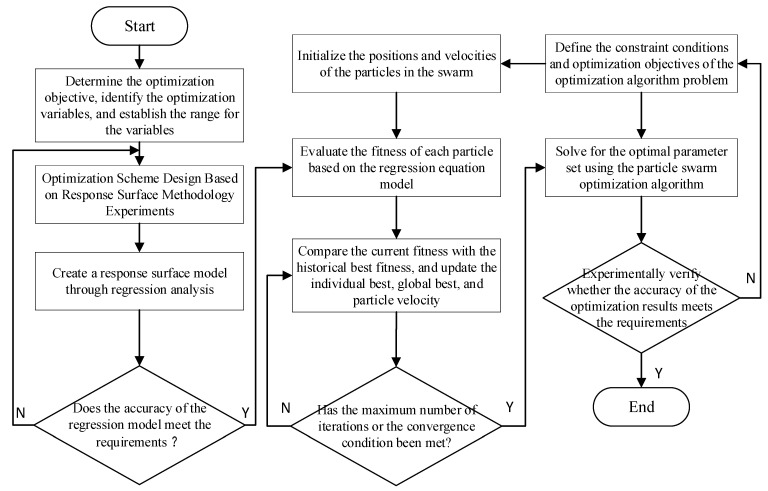
Workflow for achieving target optimization of mold operating parameters.

In this section, the particle swarm optimization (PSO) algorithm is applied to optimize the response surface equations obtained from the orthogonal experimental test data, aiming to identify parameter values that improve temperature uniformity; the combination of these two methods also demonstrates better accuracy [38]. A regression equation is derived through multiple linear regression analysis to describe the relationship between the optimization variables and objectives [39]. A predictive analysis is then conducted using the original data to test the accuracy of the regression equation. The PSO algorithm for multi-objective optimization (with temperature uniformity coefficient and thermal response rate as objectives) is enhanced using MATLAB softare R2023a (MathWorks, Natick, MA, USA). The regression equation is incorporated into the PSO algorithm to find the optimal solution, resulting in a set of optimal variables. Under these conditions, the optimization objectives are best achieved. The target optimization process flowchart is shown in Figure 10.

### 5.2. Orthogonal Experimental Design

**Figure 11:**
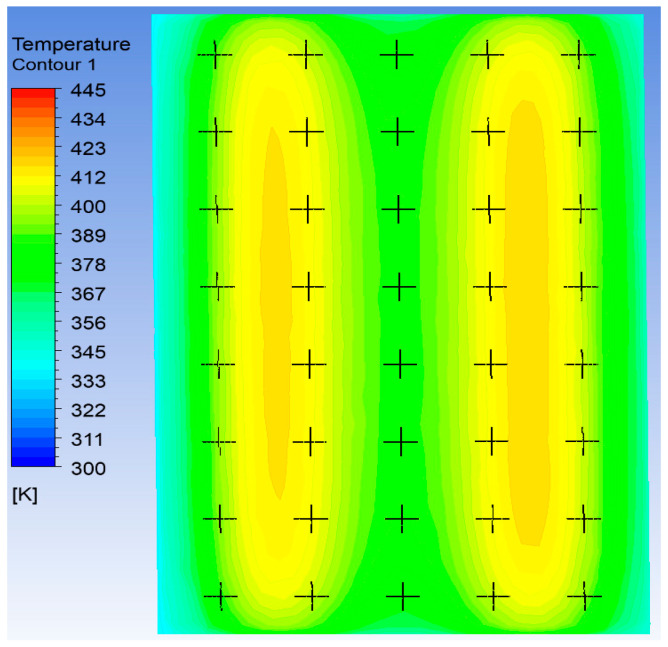
Simulation point sampling test method diagram.

In the optimization objectives, the temperature uniformity coefficient is the primary evaluation criterion, while the cavity surface thermal response rate is considered the secondary criterion. Both optimization objectives are calculated using the formula provided in Section 2.1. The temperature is determined by taking an evenly distributed array of 40 points on the cavity surface, as shown in Figure 11.

Orthogonal experiments are a method used to study combinations of multiple factors and levels. By selecting a representative subset of level combinations for experimentation, this approach significantly helps reduce the workload [40]. The model derived from the data fitting based on the orthogonal experiment design may be prone to overfitting, as it is built according to the current experimental data. This could result in poor performance on external datasets. Therefore, an external dataset is required to validate the model’s predictive capability and stability, ensuring the accuracy of the regression equation. Based on the orthogonal experimental strategy, we divided the dataset into a training set and a test set. The training set consists of 41 data points used for training and optimizing the model, while the test set includes 10 data points used for external validation. The data for both sets were obtained through orthogonal experiments with different gradients, where *q*_1_–*q*_4_, *L*_1_–*L*_4_, and *h*_1_–*h*_2_ were selected as the optimization variables. The value ranges were set as follows: heating rod power between 600 and 900 W, *L*_1_ between 20 and 30 mm, *L*_2_ between 50 and 70 mm, and *h* between 3 and 8 mm. The output optimization objective is defined as $U_{T}$ and $R_{heating}$. The training set data from the orthogonal experiment are shown in Table 4, and the test set data are shown in Table 5.

### 5.3. Response Surface Equation

In this part, the optimization objective is closely linked to multiple optimization variables. To address this, response surface methodology (RSM) is applied for regression analysis. RSM provides an effective approach for accurately predicting and optimizing the input–output relationships within engineering systems [41]. Among multivariate regression models, the full quadratic model achieves the highest predictive accuracy. Therefore, this experiment employs a full quadratic model for regression response. The regression equation for the full quadratic model is as follows [42].

(8) $$y=\beta_{0}+\beta_{1}x_{1}+\cdots +\beta_{m}x_{m}+\sum_{1\leq j,k\leq m}\beta_{jk}x_{j}x_{k}$$

The orthogonal experiment training set provides us with 41 data points based on six optimization variables and two optimization objectives. Using response surface methodology, regression response models were developed to describe the relationship of the temperature uniformity coefficient and thermal response rate with these optimization variables:

(9) $$\begin{array}{ll} y(U_{T})= & -92.5207+0.1749x_{1}-0.0039x_{2}+10.4469x_{3}-1.9599x_{4}-14.125x_{5}+3.8555x_{6}\\ & \;-0.0002x_{1}x_{2}-0.002x_{1}x_{3}+0.0004x_{1}x_{4}-0.0026x_{1}x_{5}+0.0056x_{1}x_{6}+0.0015x_{2}x_{3}\\ & \;+0.0004x_{2}x_{4}+0.0105x_{2}x_{5}-0.0128x_{2}x_{6}-0.1317x_{3}x_{4}+0.1514x_{3}x_{5}-0.3056x_{3}x_{6}\\ & \;-0.0177x_{4}x_{5}+0.0842x_{4}x_{6}-0.4703x_{5}x_{6}+0.0001{\left(x_{2}\right)}^{2}-0.0053{\left(x_{3}\right)}^{2}+0.0329{\left(x_{4}\right)}^{2}\\ & \;+0.611{\left(x_{5}\right)}^{2}+0.4403{\left(x_{6}\right)}^{2} \end{array}$$

(10) $$\begin{array}{ll} y(R_{heating})= & 0.7659+0.0016x_{1}+0.0023x_{2}+0.0009x_{3}+0.0231x_{4}-0.0182x_{5}-0.1177x_{6}-0.0001x_{1}x_{5}\\ & \;-0.0001x_{2}x_{6}-0.0011x_{3}x_{5}+0.0015x_{3}x_{6}+0.0002x_{4}x_{5}+0.0009x_{4}x_{6}+0.0017x_{5}x_{6}\\ & \;-0.0002{\left(x_{4}\right)}^{2}+0.0022{\left(x_{5}\right)}^{2}+0.0005{\left(x_{6}\right)}^{2} \end{array}$$

The regression response model achieved a root mean square error (RMSE) of 0.0121, demonstrating that the model accurately represents and predicts the data obtained from the orthogonal experiment. To further ensure the stability and accuracy of the regression equation, the predictive accuracy of the regression equation obtained from the training set is tested using the data from the test set. The predictive accuracy of the model based on the orthogonal experiment test set is shown in Table 6.

The data in the table were predicted using the response surface regression equation for both the training set and the test set. The difference between the estimated values from the regression prediction of the response objective and the predicted values is small, with error rates of 1.8% and 2.4%, respectively, indicating a high level of predictive accuracy. This suggests that the regression response model is well suited for further target optimization analysis in this experiment.

### 5.4. Target Optimization Model Design

In this section, the response surface regression equations are combined with the particle swarm optimization (PSO) algorithm to optimize the target objectives. The PSO algorithm identifies optimal solutions within a defined space by leveraging the collaborative behavior of individuals in a swarm [43]. The optimization targets are set as the cavity’s thermal response rate and temperature uniformity coefficient, with the following constraints on optimization variables: heating rod power is limited to 600–900 W, *L*_1_ to 20–30 mm, *L*_2_ to 50–70 mm, and *h* to 3–8 mm. As a result, the target optimization model can be described as follows:

(11) find qi,Li,hi∈1,2min UTs.t. Rheating≧0.72 k/s 600 w≦qi ≦900 w 20 mm≦L1≦30 mm 50 mm≦L2≦70 mm  3 mm≦L3≦8 mm

### 5.5. Analysis of Optimization Results

Following multiple iterations with the particle swarm optimization algorithm, the optimal target temperature uniformity coefficient and the corresponding optimal values for the optimization variables were obtained, as shown in Table 7. The baseline parameter settings were *L*_1_ = 25 mm, *L*_2_ = 60 mm, *h*_1_ = *h*_2_ = 5 mm, and *q*_1_ = *q*_2_ = 800 w. After optimization, the parameter set values were refined to *L*_1_ = 20.25 mm, *L*_2_ = 56.82 mm, *h*_1_ = 7.97 mm, *h*_2_ = 6.70 mm, *q*_1_ = 700.36 w, and *q*_2_ = 700.25 w.

**Figure 12:**
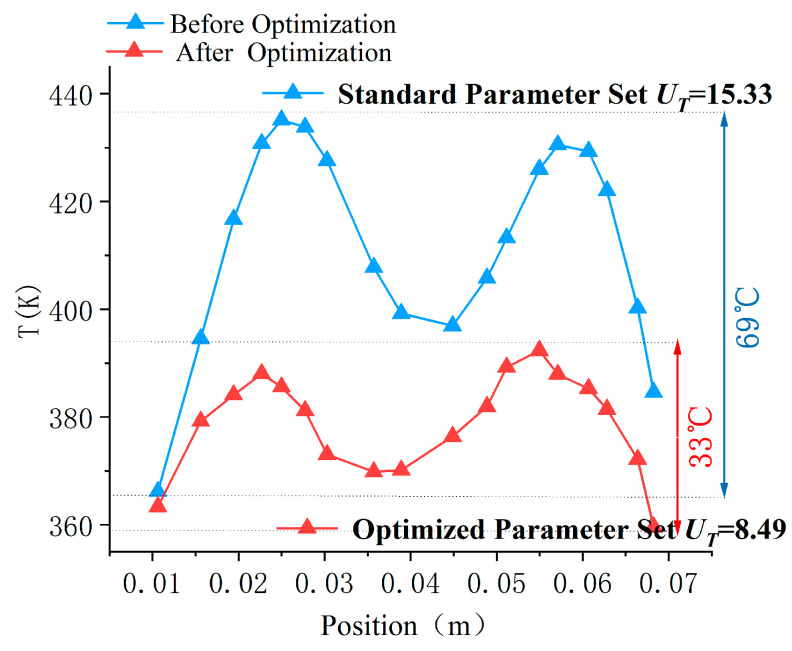
Comparison of temperature distribution between standard and optimized parameter sets.

As shown in Figure 12, the uniformity coefficient of the mold cavity surface temperature was optimized from 15.33 to 8.49, representing a reduction of 44.6%. The maximum temperature difference on the cavity surface decreased from 69 °C to 33 °C, a reduction of 52.17%, significantly enhancing the uniformity of the cavity surface temperature distribution.

Additionally, the power of the heating rod was reduced from 800 W to 700 W, resulting in a 12.5% reduction in energy consumption. At this setting, the thermal response rate of the cavity surface is 0.75 K/s, meeting the minimum requirement. This optimization not only improves the quality of the injection molding process but also reduces energy consumption.

**Figure 13:**
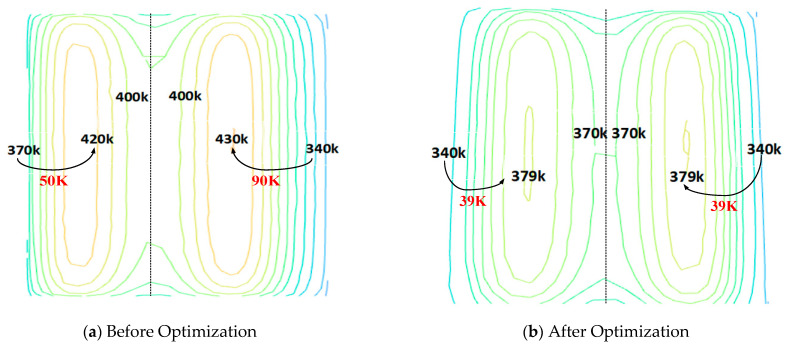
Comparison of temperature distribution and uniformity effect before and after cavity surface optimization.

Figure 13 compares the cavity surface temperature distribution before and after optimization. Before optimization, the temperature in the core region reaches 430 K, with a maximum temperature difference of 90 K, and the temperature distribution exhibits limited symmetry and uniformity. After optimization, the temperature in the core region is reduced to 379 K, the maximum temperature difference is reduced to 39 K, and the temperature distribution shows significantly improved symmetry and uniformity.

## 6. Conclusions

This study addresses the challenges of low heating efficiency and uneven temperature distribution on the cavity surface in the Rapid Heat Cycle Molding (RHCM) process. The following research and optimization improvements have been made:

(1) A numerical simulation approach was employed to study the heating process of the model, and the impact of key parameters, such as heating rod power and position, on the cavity surface temperature response rate and temperature uniformity was investigated. The results show that higher power can effectively increase the thermal response rate, but it simultaneously reduces the temperature uniformity on the cavity surface. The lateral spacing has a minimal impact on the thermal response rate, but it significantly affects temperature uniformity. Increasing the vertical distance notably improves the thermal response rate.
(2) Based on the above conclusions, an orthogonal experimental method was designed, and response surface regression equations for the thermal response rate and temperature uniformity coefficient were derived. The test results from the test set show that the average prediction errors of the equation are 1.8% and 2.4%, respectively. These equations can effectively predict the relationship between response variables and optimization objectives.
(3) Based on the above multiple regression equations, the particle swarm optimization (PSO) algorithm was used to optimize the parameters and determine the optimal parameter set. The optimization results showed that, while maintaining the required thermal response rate, the cavity surface temperature uniformity coefficient was reduced from 15.33 to 8.49, a decrease of 44.6%. The temperature difference decreased from 69 °C (with standard parameters) to 33 °C, a reduction of 52.17%, and energy consumption was reduced by 12.5%.

This demonstrates that the use of particle swarm optimization to optimize the factors influencing temperature distribution has significantly improved the temperature uniformity on the mold cavity surface, providing a solid foundation for enhancing product quality in RHCM technology. This study focuses on the optimization analysis of the electric heating rod mold heating method, which is widely used in engineering applications. The proposed optimization approach is highly versatile and can be extended to the simulation of heating systems in other complex systems. However, the research also has some limitations: since the simulation assumes uniform thermal conductivity and a symmetric geometric mold, challenges may arise when dealing with complex geometries and material distributions. As a result, the applicability and accuracy of the model may be affected. Future research could focus on addressing these challenges by adapting the optimization method to accommodate complex geometries and different injection molding materials. This could involve the introduction of more precise numerical solutions, improvements to the model, or enhancing adaptability under various working conditions by adjusting boundary conditions and optimization algorithms.

## Figures and Tables

**Table 1:** Material parameters for the mold and heating rod.

Component	Material	*λ*	*C*	*ρ*
Mold	Cr12Mo1V1	45	500	7833
Heating Rod	MgO-SiO_2_	5.5	1100	2700

**Table 2:** Mold operating parameter simulation design plan.

	Horizontal Distance *L* (mm)		Vertical Distance *h* (mm)		Heating Rod Power
Serial No.	*L*_1_	*L*_2_	*h*_1_	*h*_2_	*p*
1~4	15/20/25/30	80	12	12	600 w
5~8	15	50/60/70/80	12	12	600 w
9~12	15	80	3/6/9/12	3/6/9/12	600 w

**Table 3:** Impact of changes in operating parameters on cavity thermal response rate.

Test Serial No.	Operating Parameter Set				Cavity Thermal Response Rate	
	*L*_1_	*L*_2_	*h*_1_	*h*_2_	Increase (k/s)	Growth Rate
1~4	15–30	80	12	12	0.04	14%
5~8	15	50–80	12	12	0.14	52%
9~12	15	80	3–12	3–12	0.86	150%

**Table 4:** Orthogonal experiment training set data table.

No.	Optimization Variables						Optimization Objectives	
	Power (W)		Position Parameters (mm)				TRR (k/s)	TUC
	*q*_1_	*q*_2_	*L*_1_	*L*_2_	*h*_1_	*h*_2_	*R_heating_*	*U_T_*
1	600	600	20	50	3	3	1.028	18.96
2	700	700	20	55	5	5	0.955	12.13
3	800	800	20	60	7	7	0.85	12.74
4	900	900	20	65	8	8	0.794	17.81
5	700	800	22	50	8	3	1.075	32.82
6	600	900	22	55	7	5	0.968	24.66
7	900	600	22	60	5	7	0.939	26.30
8	800	700	22	65	3	8	0.917	34.11
9	800	900	24	50	5	3	1.357	38.80
10	900	800	24	55	3	5	1.332	28.40
11	600	700	24	60	8	7	0.679	11.17
12	700	600	24	65	7	8	0.645	13.62
13	900	700	26	50	7	3	1.172	35.47
14	800	600	26	55	8	5	0.813	23.75
15	700	900	26	60	3	7	1.049	24.38
16	600	800	26	65	5	8	0.738	15.16
17	600	600	28	70	3	3	0.79	22.12
18	700	700	28	70	5	5	0.786	19.06
19	800	800	28	70	7	7	0.749	16.38
20	900	900	28	70	8	8	0.77	16.20
21	600	700	30	50	7	8	0.781	27.69
22	700	600	30	55	8	7	0.757	20.18
23	800	900	30	60	3	5	1.269	31.73
24	900	800	30	65	5	3	1.11	30.67
25	700	800	20	50	3	5	1.18	21.47
26	600	900	20	55	5	3	1.139	29.91
27	900	600	20	60	7	8	0.787	20.68
28	800	700	20	65	8	7	0.693	15.78
29	800	600	22	50	5	7	0.946	25.74
30	900	700	22	55	3	8	1.134	35.51
31	600	800	22	60	8	3	0.906	29.32
32	700	900	22	65	7	5	0.872	23.38
33	600	600	24	70	3	5	0.713	24.63
34	700	700	26	70	5	3	0.822	20.34
35	900	900	28	50	8	5	1.185	40.05
36	800	800	28	55	7	3	1.099	44.22
37	700	700	28	60	5	8	0.844	19.07
38	600	600	28	65	3	7	0.776	21.79
39	800	900	30	70	7	8	0.764	17.41
40	900	800	20	70	8	7	0.697	21.44
41	600	800	22	70	5	8	0.621	18.13

**Table 5:** Orthogonal experiment test set data table.

No.	Optimization Variables						Optimization Objectives	
	Power (W)		Position Parameters (mm)				TRR (k/s)	TUC
	*q*_1_	*q*_2_	*L*_1_	*L*_2_	*h*_1_	*h*_2_	*R_heating_*	*U_T_*
1	650	650	23	56	4	4	0.979	14.86
2	650	750	26	62	6	6	0.841	13.35
3	750	650	26	68	6	4	0.785	20.95
4	750	750	23	56	6	4	1.031	17.92
5	850	850	29	62	4	4	1.181	27.66
6	850	650	29	68	6	6	0.774	19.37
7	650	850	23	68	6	6	0.794	22.77
8	750	750	29	56	4	6	1.079	28.26
9	850	750	26	68	4	4	0.988	24.66
10	750	850	26	56	6	4	1.137	30.78

**Table 6:** Orthogonal experiment test set prediction accuracy table.

	Optimization Variables						Actual Value	Predicted Value	Accuracy
Group	*L*_1_	*L*_2_	*h*_1_	*h*_2_	*p*_1_	*p*_2_			
1	20	50	3	3	600	600	18.96	19.89	95.3%
2	24	50	5	3	800	900	38.80	38.65	99.6%
3	20	50	3	3	600	600	1.028	1.026	99.8%
4	20	55	5	5	700	700	0.955	0.952	99.7%

**Table 7:** Comparison between optimized and standard parameter sets.

	Optimization Variables						Optimization Objective
	Power (W)		Position Parameters (mm)				Coefficient
	*q*_1_	*q*_2_	*L*_1_	*L*_2_	*h*_1_	*h*_2_	*U_T_*
Standard Parameter Set	800	800	25	60	5	5	15.33
Optimized Parameter Set	700	700	20	56.8	8	6.7	8.49

## Data Availability

The original contributions presented in this study are included in the article. Further inquiries can be directed to the corresponding author.
